# Supplementary material for: Is women’s household decision-making autonomy associated with their higher dietary diversity in Bangladesh? Evidence from nationally representative survey
Source: PLOS Glob Public Health. 2023 Jul 19;3(7):e0001617. doi: 10.1371/journal.pgph.0001617 (PMC10355378; doi:10.1371/journal.pgph.0001617)
Supplement: Attachment — Submitted filename: final resoponse letter.docx [file pgph.0001617.s001.docx]

**Response letter**

The manuscript was modified according to the reviewer comments.

**Journal Requirements:**

1. In the online submission form, you indicated that "The data that support the results of this research are available from the DHS website (https://www.dhsprogram.com/), but they are subject to restrictions since they were utilized under license for the present study and are thus not publicly available. Data are however available upon reasonable request.". All PLOS journals now require all data underlying the findings described in their manuscript to be freely available to other researchers, either 1. In a public repository, 2. Within the manuscript itself, or 3. Uploaded as supplementary information.

This policy applies to all data except where public deposition would breach compliance with the protocol approved by your research ethics board. If your data cannot be made publicly available for ethical or legal reasons (e.g., public availability would compromise patient privacy), please explain your reasons by return email and your exemption request will be escalated to the editor for approval. Your exemption request will be handled independently and will not hold up the peer review process, but will need to be resolved should your manuscript be accepted for publication. One of the Editorial team will then be in touch if there are any issues.

**Response:**

**Revised as -** The data that support the results of this research are available from the DHS website (https://www.dhsprogram.com/), but they are subject to restrictions since they were utilized under license for the present study and are thus not publicly available. Data are available upon reasonable request after getting the approval from DHS under Bangladesh section (email: info@dhsprogram.com).

**Additional Editor Comments (if provided):**

This paper is of global and national importance. Several papers are published on similar issue using DHS data or other sources of panel data. Less references are noted on the relevant topic. Incorporating comments of the two reviewers is likely to improve the quality.

**Response:** *Thank you for your suggestion. We already added some other references to the introduction section of the revised manuscript (please see the line no: 77-89).*

**Reviewer 1: Minor revision**

The article is nicely written and in well described scientific fashion. Few of the grammar should be revised by proof reading. Though the methodology section is clearly written, it provides ambiguity if it is original article or a secondary analysis. Please write how the data has been extracted and used to conduct the present study.

**Response:** *We used data from Demographic and Health Surveys (DHS), which were nationally-representative household surveys implemented in over 90 countries by ICF international under the financial support of the United States Agency for International Development to understand health and population trends in developing countries (https://dhsprogram.com/). We obtained approval from ICF International and downloaded anonymous datasets of Bangladesh demographic and health survey 2014.*

*We revised it in the methodology section (please see line no: 121-126)*

All the participants in this study were ever married women aging 15 to 49-years-old, among which married /living together (n= 16,858) and divorced/ separated/ widowed (n= 1,005). A secondary data assessment was performed to examine the association of dietary diversity with the potential socioeconomic factors and women’s decision-making autonomy with their complete dietary details (n = 17,842). We deleted 21 participants due to the presence of missing values.

**Reviewer 2: Major revision**

**Comment 1.** Title: Suggest that the authors consider revising the title: “Is women’s household decision-making autonomy associated with their higher dietary diversity in Bangladesh? Evidence from nationally representative survey”- I suggest bringing the decision-making autonomy before the DDS.

**Response:** *Thank you for your suggestion. I already revised the title according to your suggestion (please see line no.: 1-3).*

**Comment 2.** Line 29-32: Women who had higher and secondary education were 2.72 (95% CI: 1.49-3.02; p = 0.025) and 1.31 (95% CI: 0.58-2.18; p = 0.029) times more likely to achieve higher DD than uneducated women, as well as rich women (OR 6.49; 95% CI: 4.12-8.5; 32 p = 0.037) than poor women.

- Suggest revising the language: instead of uneducated, apply women with no education; instead of rich women, apply women in the richest quintile compared to women in the lowest quintile.

**Response:** *The language has been revised according to the suggestion (please see line no.: 31-32).*

**Comment 3.** Line 52-43: Despite evidence of escalating cases of malnutrition, the underlying causes of a low quality, monotonous diet and the role of women are poorly understood.

- This is not very clear. “The role of women” is a bit vague. I suggest clarifying this line or remove this line altogether.

**Response:** *I already revised the mentioned line as*

*low-quality monotonous diet is regarded as a major factor of malnutrition among women. While there is mounting evidence of an increase in malnutrition, the underlying causes of the women's poor dietary intake remain unclear* *(Please see line no: 54-56)*

**Comment 4.** Line 56-59: Moreover, the monotonous consumption of starchy staple foods and other foods with lower nutritional quality is one of the main reasons for the micronutrient deficiency in individuals, especially in pregnant women and lactating mothers [9]. Acute scarcity of micronutrients increases the death rate of the childbearing mother as well as the child [9].

- Please check the reference “9”. The article that you are referring to here is actually a very similar article as yours conducted in a different country. I suggest that you add the original articles that the Ghana authors have cited on micronutrient deficiency along with this one.

**Response:** *The original articles on micronutrient deficiency have been added as reference 10 and 11 (please see line no 61-62).*

**Comment 5.** Line 62-paragraph starting in line 62: please separate the sections on DD and women.

**Response:** *The sections on DD and women empowerment have been separated (line no 70).*

**Comment 6.** Line 66-77: The literature review in this section should include the Ghana paper on women’s household decision making and DD (ref 9), and the Sinharoy paper (ref 3) and other works in the related areas in other low and middle income countries.

**Response:** *We modified the introduction section according to the reviewer’s suggestion by adding some findings from previous studies where the relationship between women’s decision-making and empowerment, and diet diversity (DD) reported in low and middle-income countries has been discussed (please see the line no: 77-89).*

Other relevant studies emphasized the relationship between woman empowerment and child nutrition [19] and diet diversity [20]. Women’s participation in decision-making regarding household purchases was significantly associated with the attainment of higher DD in Ghana [8, 21] and in sub-Sahara African countries the women with higher decision-making autonomy had access to diversified foods [22]. In Bangladesh it was evident that schooling of women was positively associated with DD score in rural areas [3]. Higher dietary diversity score was reported in case of empowered women and their children in Timor-Leste [23]. A similar result was also observed in the case of women living in rural Kenya [24]. In the western hilly region of Nepal women with job and adequate nutrition knowledge belonging to wealthier households, joint families had higher odds of dietary diversity [25].

One previous study reported a promising relationship between woman empowerment and household dietary diversity of the Habiganj district of Bangladesh, but it was not a country representative study [3].

**Comment 7.** Line 75-77: However, additional research is required to provide a more comprehensive knowledge of the association between diet diversity and women's autonomy in various circumstances.

- The authors clearly need to mention that similar works have been done in other context, but, not in Bangladesh. As such, this work is important to add to the body of literature.

**Response:** *I already revised the manuscript following the reviewer’s suggestion. (Please see the line no: 89-93)*

Similar other studies were also conducted elsewhere to examine the relationship of women’s dietary diversity, socioeconomic conditions, and their decision-making autonomy in another context but, not in Bangladesh. To the best of our knowledge, this current study is the first study based on country representative data in Bangladesh. As such, this work is important to add to the body of literature.

**Comment 8.** Line 110-115: Among the 17,300 households, 17863 ever married women were interviewed. A secondary data assessment was performed to examine the dietary diversity and differentials. The participants in this sample were women aged 15 to 49- years-old (n = 17,842) with their complete dietary details. The following parameters were used in bivariate and multivariate analysis to assess population characteristics and analyze correlations between dietary diversity and educational qualification or place of residence.

- Please clarify in the sample if all participants were ever married or women of reproductive health.

**Response:** *We modified this sentence according to the reviewer’s suggestion. (Please see line no: 121-126)*

*We add:* All the participants in this study were ever married women aging 15 to 49-years-old, among which married /living together (n= 16,858) and divorced/ separated/ widowed (n= 1,005).

- Please clarify what is meant by differentials

**Response:** *We already modified the sentence (please see line no: 121-126) as-*

A secondary data assessment was performed to examine the association of dietary diversity with the potential socioeconomic factors and women’s decision-making autonomy with their complete dietary details (n = 17,842)

- Analyze correlation between these two variables (DD and education/ place of residence)?

**Response:** *We analyzed the odds between the variables by using binary logistic regression. And the ODDS ratio between DD and education was 2.72 and 1.31 for the women having higher education and secondary education, respectively. In bivariate analysis, the women living in urban areas had 1.69 times higher diet diversity score comparative to the women living in rural areas. The line is removed from the section as it is described in statistical analysis section (please see line no: 179-182).*

The relationship between DD and women’s decision-making autonomy (viz., spending money, making household purchases, and own health care) along with other demographic characteristics was determined using bivariate and multivariate logistic regression, and the result was reported as the odds ratio*.*

**Comment 9.** Line 132-139: In this study, we considered the women’s decision-making autonomy based on whether they could participate in the decision-making of (i) spending money for the household, (ii) household purchases, and (iii) own health care, which may also be considered as a representative variable of woman empowerment. We re-coded the variables ‘final say on deciding what to do with the money earned by the husband’ and ‘who decides how to spend money in the household’, from the dataset of BDHS 2014, into ‘whether the women could participate in the decision of spending money’. We also re-coded the variable ‘final say on making large household purchases’ and ‘final say on making purchases for daily needs’ from the parent data set into the variable ‘whether the women can participate in the decision-making of household purchases’

- It would be interesting if the authors explain why decision making about small purchases and large purchases are grouped together. Also, please clarify how spending money for household different from the household purchases.

**Response:** *We merged small purchases (such as purchasing daily commodities including foods, medicines) and larges purchases (sofa, freeze, kitchenware etc.) together to understand the association of overall purchasing ability with women’s diet diversity score. (Please see line no: 152-154).*

We merged the decision on small purchases and larges purchases together to understand the association of women’s overall purchasing ability with their diet diversity score.

*Spending money for households (for example child education, childcare, visits to their family or relatives etc.) are different from household purchases where the money spends for purchasing any goods or product (Please see line 154-156)*

Decision of spending money for households (viz., child education, childcare, visits to their family or relatives etc.) were different from the decision of household purchases where the money spends for purchasing any goods or products.

**Comment 10.** Line 144: Quintile from poorest to richest. However, table 1 indicates that the authors have collapsed two groups. Please clarify that in the text.

**Response:** *We merged poorest and poorer to poorer as the number of respondents in poorest quantile is lower and to avoid the zero-sell count during the analysis. (Please see line no: 162-163)*

**Comment 11.** Line 159: Statistical analysis

- DHS applies multistage cluster sampling. It is expected that analyses of DHS data apply “SVY” commands to address the clustering effect. Please clarify this if this approach has been adopted.

**Response:** *We do not use SVY comment for fits statistical models for complex survey data as it is a secondary data analysis. We addressed these limits in the strength and limitation section. (Please see line no: 307-308).*

**Comment 12.** Discussion: The discussion section should highlight the limitations and weaknesses of the study in relation its strength. This is a cross sectionals study, and the issues of cross-section data should be discussed. Also, some of the variables that the authors considered as independent (economic status, education, job status, purchasing decision) can be collinear to each other. That has also implications for the results. Please discuss limitations.

The authors can also discuss that this particular construct of autonomy and it's pathway to attaining the outcome are complex, and future research is also necessary.

**Response:** *We added some strength and limitation in discussion section. (Please see line no: 300-311****).***

The data quality in this study is fairly strong since it is based on a nationally representative survey that is supervised and controlled by an international expert committee. As far our knowledge, this is the first study based on country representative data in Bangladesh which revealed the association of women’s decision-making autonomy, and socioeconomic factors with the attainment of dietary diversity. The outcomes from nationally representative data are more reliable to policymakers. Still, there are some limitations to this research that should be considered. The dataset utilized in this research is derived from a cross sectional study, BDHS 2014 [26]. We did not use SVY comment for fits statistical models for complex survey data as it is a secondary data analysis. Also, some of the variables that we considered as independent (economic status, education, job status, purchasing decision) can be collinear to each other which may have some implications in the results.

**Inventory file**

Abstract

**Comment 1:** In line no 17-18 it is suggested to use term mothers and their children.

**Response:** *We already modified the word. (Please see line no 18)*

**Comment 2:** Please use the full form of DDS before using the abbreviation.

**Response:** *We already added the full form of DDS in the manuscript. (Please see line no: 23)*

**Comment 3:** Specify the selection criteria/ sampling strategy

**Response:** *We used data from Demographic and Health Surveys (DHS), which were nationally representative household surveys implemented in over 90 countries by ICF international under the financial support of the United States Agency for International Development to understand health and population trends in developing countries (https://dhsprogram.com/). We obtained approval from ICF International and downloaded anonymous datasets of Bangladesh demographic and health survey 2014.*

*We revised it in the methodology section (please see line no: 121-126)*

All the participants in this study were ever married women aging 15 to 49-years-old, among which married /living together (n= 16,858) and divorced/ separated/ widowed (n= 1,005). A secondary data assessment was performed to examine the association of dietary diversity with the potential socioeconomic factors and women’s decision-making autonomy with their complete dietary details (n = 17,842). We deleted 21 participants due to the presence of missing values.

**Comment 4:** Same format of writing is recommended in the results section to indicate the Odds ratio and CI; please review it.

**Response:** *We already modified the error formats in result section.*

**Comment 5:** Please include one line of recommendation from the study in the conclusion section of abstract.

***Response:*** *We included the recommendation from this study in the conclusion section of abstract. (Please see line no: 34-37)*

*This study highlighted the association of several socioeconomic conditions of ever married women and their dietary diversity score in Bangladesh. Therefore, promoting the women’s education status, improving the socioeconomic conditions, and prioritizing their decisions are recommended for the attainment of higher dietary diversity score.*

**Comment 6:**

Introduction section

Well presented background and specified gap identification. Please reveal the relationship between woman empowerment and household dietary diversity as indicated in line 80-82. Also include if the study has shown any relation between the women’s autonomy and dietary diversity.

**Response:**

*We modified the introduction section according to the reviewer’s suggestion by adding some findings from previous studies where the relationship between women’s decision-making and empowerment, and diet diversity (DD) reported in low and middle-income countries has been discussed. (Please see the line no: 77-89)*

**Comment 7:**

Methodology

The methodology section is well written. It clearly expresses the strategy used in the BDHS. I hope the current study has been conducted by extracting the secondary data from the survey. I would like to suggest the authors to also clearly mention what strategy was used to extract the required data and how was the data used to perform the current study.

**Response:** *We used data from Demographic and Health Surveys (DHS), which were nationally representative household surveys implemented in over 90 countries by ICF international under the financial support of the United States Agency for International Development to understand health and population trends in developing countries (https://dhsprogram.com/). We obtained approval from ICF International and downloaded anonymous datasets of Bangladesh demographic and health survey 2014.*

*We revised it in the methodology section (please see line no: 121-126)*

All the participants in this study were ever married women aging 15 to 49-years-old, among which married /living together (n= 16,858) and divorced/ separated/ widowed (n= 1,005). A secondary data assessment was performed to examine the association of dietary diversity with the potential socioeconomic factors and women’s decision-making autonomy with their complete dietary details (n = 17,842). We deleted 21 participants due to the presence of missing values.

**Comment 8:**

Results

The results are well explained. Please review the table 1 and mention Mean ± SD / % in the rows as required. Please review the journals criteria for labeling the tables from the results section if required.

**Response:** Revised accordingly.
