# Supplementary material for: Is women’s household decision-making autonomy associated with their higher dietary diversity in Bangladesh? Evidence from nationally representative survey
Source: PLOS Glob Public Health. 2023 Jul 19;3(7):e0001617. doi: 10.1371/journal.pgph.0001617 (PMC10355378; doi:10.1371/journal.pgph.0001617)
Supplement: Attachment — Submitted filename: reviewer coments.docx [file pgph.0001617.s002.docx]

**Response letter**

Reviewer 1

**Comment 1.** Line 25: “was” should be “were”

**Response:** *Thank you for your suggestion. We have already revised this error.*

**Comment 2.** Lines 29=8-29: “Using logistic regression, the odds”. Odds of what? Re-write for clarity.

**Response:** *Thank you for your suggestion. We have already rewritten this sentence (please see line no:28)*

**Comment 3.** The conclusion refers to the study population as ever-married women but in the methods of the abstract, this is not stated.

**Response:** *We added this in abstract section (please see line no: 21).*

**Comment 4.** The use of the abbreviations “DD” and “DDS” is confusing. I suggest the authors should stick with only one, perhaps DDS which is more popular in literature.

**Response:** *We revised the whole manuscript according to your suggestion. We replace all DD with DDS.*

**Comment 5.** Why re-group the food groupings into 9 instead of 10 the food group indicator, validated for women of fertile age (Minimum Dietary Diversity for Women (MDD-W**))**

**Response:** *In this study, two food groups (other fruits, other vegetables) were regrouped into one outcome variable into other fruits and vegetables. The number of observations was small for other vegetables. Moreover, 9 food groups were recommended by USAID and FAO [1, 2]. We followed the method recommended by the USAID [3]. Moreover, a recent study by* Martin-Prevel et al. (2017) *shows that the food group indicator (FGI-9) and FGI-10, performed similar results [4].*

**Comment 6.** Both the titles of the manuscript and the aim of the study (to examine the relationship between women’s decision-making autonomy and their attainment of higher DD) suggest that the statistical analysis should focus on hypothesis testing. However, the authors seemed to have employed an explorative method in the data analysis to identify the factors associated with DDS. The title and objective can be revised to reflect the analysis, or the analysis should be re-examined.

**Response:** *I think the reviewer is a little bit misleading here. Usually, the hypothesis is tested by ‘yes’ or ‘no’. But we employed regression analysis to find out the confounders associated with women’s decision-making autonomy, emphasizing the women’s dietary diversity. Therefore, in objectives we mentioned not only women’s dietary diversity but also include some sociodemographic factors as well. For more clarification, we copied here the objective of our study: (please see line no: 96-99)*

*This study aimed to examine the relationship between women’s decision-making autonomy and their attainment of higher DDS, as well as some socio-demographic variables that can independently predict women’s attainment of higher DDS using the country representative data collected from the Bangladesh Demographic and Health Survey.*

**Comment 7.** A description of the data analysis is also scanty and a bit more detail may be necessary to understand how the analysis was done. For example, what criteria qualified a variable to be included in the multivariate analysis? And how did the authors examine multicollinearity between explanatory variables?

**Response:** *In* *this study, the explanatory variables were selected based on previous literature that shows relation with the women’s decision-making process that area available in the data set. we revised and added it in line 148-151. To minimize the multicollinearity effect, we tested the variance inflation factor and found that it was less than 10 in all cases (please see line no:* *148-151).*

**Comment 8.** Lines 211-213: sentence structure needs clarity in writing.

**Response:** *Revised accordingly (please see line no: 221-223)*

*The results of bivariate and multivariate analysis, which report the odds between women’s diet diversity and associate covariates with women's decision-making autonomy are presented in* ***Table 2.***

**Comment 9.** Lines 217-218: it is better to report the results by giving direction to the association e.g., residing in an urban area compared to a rural area was associated with a higher DDS; etc.

**Response:** *We revised* *accordingly.*

**Comment 10.** The 95% C. I for secondary in the educational status doesn’t seem to agree with the p-value (Table 2), please re-examine this.

**Response:** *We rechecked, and the result find ok.*

**Comment 11.** Lines 301: The phrase “fairly strong” seems a bit strange. Perhaps, we should discuss the generalizability of the results for all women of reproductive age in Bangladesh.

**Response:** *The phrase “fairly strong’’ has been replaced by “almost well”. (Please see line no. 332)*. *The data quality in this study is* *almost well since it is based on a nationally representative survey that is supervised and controlled by an international expert committee.*

*This study encompasses only the data of ever married women aged 15–49 years old. Therefore, generalizability of the results for all women of reproductive age in Bangladesh is not possible. We added it in limitation section (please see line no: 340-342)*

**Comment 12.** Lines 309-311: collinearity can be examined and handled in the data analysis step. Please see my comment there

**Response:** *To minimize the multicollinearity effect, we tested the variance inflation factor and found that it was less than 10 in all cases. We mentioned it in line 150-152.*

**Reviewer 2**.

**Comment 1:** In line 112 you have mentioned that the questionnaire was adopted whereas in Later portion in the line 117 you had written as questionnaire was developed. This has created ambiguity in validity of questions. How do you address this comment?

**Response:** *Thank you for your suggestion. Actually, the questionnaire was adopted in English and then translated into Bangla.*

*We revised the manuscript to remove ambiguity (please see line no: 119-120).*

*The questionnaire is adopted for use in Bangladesh through a series of meetings with a Technical Working Group (TWG) that consisted of representatives from NIPORT, Mitra and Associates, International Center for Diarrheal Disease Research, Bangladesh (ICDDRB), USAID/Bangladesh, and ICF International. Draft questionnaires were then also circulated to other interested groups and were reviewed by the 2014 BDHS Technical Review. The questionnaire was adopted in English and then translated into Bangla, the national language of Bangladesh. Experts and volunteers reviewed the translations, and to validate the questionnaire, a pilot study was conducted.*

**Comment 2:** Line 163- avoid the zero-sell count is wrong please write avoid the zero-cell count

**Response:** *Revised accordingly (Please see line no.: 171).*

Ethical consideration

**Comment 3:** Were any of the participant data had to be omitted because the participant had refused for using his/her data consent for further analysis process.

**Response**: *This study is based on secondary data analysis and the data set encompasses no available information on it*.

Results

**Comment 4:** In Table 2 how have you put significant values * and **? Please explain in response of this comment.

**Response:** *The level of significance was mentioned under the table (please see line no: 245).*

*Significant value *p <0.05, **p <0.01*

*These were used to denote the strength of the statistical findings. The significance level is a measure of the probability that the observed results are due to chance. In most cases, a significance level of 0.05 (or 5%) is used, indicating that there is a 5% or less chance that the observed results occurred by random chance alone. When the p-value (the measure of statistical significance) is below this threshold, it is considered statistically significant.*

*Here's how the asterisks are typically used:*

*Single asterisk (*): It is commonly used to denote a p-value less than 0.05 but greater than 0.01. For example, p < 0.05 would be denoted as *, indicating statistical significance at the 5% level.*

*Double asterisks (**): They are used to denote a p-value less than 0.01, indicating higher statistical significance. For example, p < 0.01 would be denoted as **.*

**Comment 5:** Line 253 to 262 the results have only suggested to improve the women’s decision making but not any strategies. Please suggest some successful strategies in reference to relevant literatures.

*Response: Revised accordingly. please see the line number: 313-330*

**Comment 6:** Line 309 to 311 are there any strategies applied to address the mentioned limitations during analysis. If any please explain.

***Response:*** *To minimize the multicollinearity effect, we tested the variance inflation factor and found that it was less than 10 in all cases. We mentioned it in line 150-152.*

**Reference:**

1.	USAID, Maternal dietary diversity and the implications for children’s diets in the context of food security. 2012, USAID Washington.

2.	FAO, Guidelines for measuring household and individual dietary diversity. Version 2, (2007), <http://www.fao.org/3/a-i1983e.pdf>.

3.	USAID, Multi-sectoral nutrition strategy 2014–2025. 2014, USAID Washington, DC.

4.	Y. Martin-Prevel, M. Arimond, P. Allemand, D. Wiesmann, T.J. Ballard, M. Deitchler, M.C. Dop, G. Kennedy, A. Lartey, W.T.K. Lee, and M. Moursi, Development of a Dichotomous Indicator for Population-Level Assessment of Dietary Diversity in Women of Reproductive Age, Current Developments in Nutrition. 1 (2017), cdn.117.001701, <https://doi.org/10.3945/cdn.117.001701>.
